# Communication and fear of cancer recurrence in colorectal cancer survivors and their partners

**DOI:** 10.1007/s00520-025-09630-3

**Published:** 2025-06-16

**Authors:** Hannah Z. Catzen, Paul Abrahamse, Kevin C. Ward, Sarah T. Hawley, Christine M. Veenstra

**Affiliations:** 1 University of Michigan, Ann Arbor, MI USA; 2 Emory University, Atlanta, GA USA

**Keywords:** Coping, Communication, Cancer, Partners, Fear

## Abstract

**Purpose:**

Little is known about how colorectal (CRC) survivors and their partners communicate about cancer or experience fear of cancer recurrence (FCR). To effectively support and guide survivors and partners, we designed a dyadic survey and investigated whether survivors’ health-related quality of life (HRQoL) was associated with dyadic discordance in communication or FCR.

**Methods:**

From 2019–2020 we surveyed survivors of stage III CRC and their partners. We measured dyadic cancer communication using the Lewis Mutuality and Interpersonal Sensitivity Scale. We asked dyads 3 questions about FCR. We measured survivors’ HrQoL using the PROMIS-29 + 2 profile, v 2.1. We used bivariate analyses and multivariable logistic regressions to compare survivor and partner responses, describe characteristics of discordant dyads, and assess associations between survivors’ HrQoL and discordance in cancer communication and FCR.

**Results:**

We analyzed data from 307 paired survivor-partner dyads (51% survivor response rate; 73% partner response rate). 29% dyads were discordant in cancer communication. 26% dyads were discordant in FCR. Greater discordance in cancer communication was associated with female survivor sex and greater partner educational attainment. Greater discordance in FCR was associated with younger survivor age and receipt of radiation (all *p* < 0.05). Worse survivor HRQoL was associated with greater discordance in FCR and receipt of radiation (all *p* < 0.01).

**Conclusions:**

Our findings highlight the complex dynamics dyads experience during CRC survivorship. While open communication is crucial, differing levels of shared fears and concerns can significantly impact health-related quality of life (HRQoL). Addressing these issues, especially FCR, can enhance patient-centered survivorship care.

## Introduction

Colorectal cancer (CRC) is the third most common cancer diagnosis in men and women in the United States. With advances in treatment the population of CRC survivors in the US is greater than 1.5 million people and continues to grow [1]. While many long-term CRC survivors report health-related quality of life that is similar to that of the general population, a substantial proportion of CRC survivors report decreased health-related quality of life (HrQoL). Physical and psychosocial concerns including neuropathy, bowel dysfunction, pain, fatigue, insomnia and depression have been described years after diagnosis [2, 4].

Fear of cancer recurrence (FCR) is a common issue among cancer survivors [5] that can negatively impact their quality of life [6, 7]. Among CRC survivors in particular, FCR can be triggered by frequent medical tests and appointment that occur during the five-year regimen of surveillance for cancer recurrence after the completion of curative-intent disease [7]. Importantly, FCR is not limited to survivors alone; partners and caregivers often report FCR levels that are equal to or even exceed those of survivors themselves, indicating a profound psychological burden shared within the dyad [8]. Some studies have suggested that in couples, FCR experienced by one partner can influence that of the other, which has important implications for communication between survivors and their partners [9, 12].

Existing research highlights that effective dyadic communication, characterized by shared or mutual understanding, can reduce decisional conflict, increase HrQoL, and enhance relationship functioning between patients with cancer and their partners [13, 14]. This type of communication, known as open or concordant communication, has been associated with improved dyadic coping and resilience [15, 16]. In contrast, discordant communication, defined as lacking openness and mutual understanding, has been associated with worse patient and partner depression and anxiety, and with lower relational satisfaction [16, 17].

While there is a significant body of research on patient-caregiver dyadic communication and patient and partner FCR, little is known about how survivors of CRC communicate with their partners about their cancer or about how well they align in their fears about the cancer or about cancer recurrence. To address these gaps, we conducted a dyadic survey study of CRC survivors and their partners to assess communication about cancer and FCR. We sought to quantify discordance between survivors and their partners in their communication about the cancer and about FCR, and to describe characteristics of survivor-partner dyads who are discordant in communication and FCR. We hypothesized that survivors’ HRQoL is negatively associated with discordance in cancer communication and FCR. The overarching goal of this study is to better understand how clinicians can support and advise both CRC survivors and their partners about communication and FCR during the survivorship period and to inform future dyadic interventions to enhance patient-centered survivorship care.

## Methods

### Study design, setting, and participants

As previously described [18, 19], we conducted a cross-sectional dyadic survey study of CRC survivors and their partners. We identified survivors aged 21–85 with surgically resected, pathologic Stage III colon or rectal cancer diagnosed 2014–2018. Survivors were identified via the tumor registries of the University of Michigan (Ann Arbor, Michigan) and the Billings Clinic (Billings, Montana), and via archival registry data from the Georgia Cancer Registry, which includes patients from across the state of Georgia.

Exclusion criteria included metastatic cancer (Stage IV) at diagnosis, identifiable cancer recurrence in the years between completion of curative-intent therapy and receipt of survey, and death prior to survey deployment. Partners (spouse, domestic partner, significant other) living in the same household as the patient—as identified by the patient—were eligible. We distributed surveys between April 2019 and February 2020 using a modified multimodal Dillman approach [20] to invite survivors and partners to participate and monitor and track survey responses. To bolster survey response rates, survivor non-respondents were re-contacted via postal mail and telephone and were given the option to complete the survey on paper or verbally over the phone. Eligible survivors were mailed a patient survey packet with a $10 cash gift and a separate partner survey packet to give to their partner. Survivors and partners returned their completed surveys in separate envelopes. Upon receipt of their completed survey, partners were mailed a $10 cash gift. Completed surveys from survivors and partners were linked using unique identification numbers. We performed extensive data checks of completed surveys for logic, errors, and omissions, and contacted participants as needed to obtain missing information. The study protocol was approved by the Institutional Review Boards of the University of Michigan, the Billings Clinic, Emory University, and the State of Georgia Department of Public Health. The institutional review boards at all study sites approved this study and waived the need for written informed consent. Return of a completed survey was considered to be implied consent to participate. Patients and partners who did not wish to participate were free to choose not to complete or return a survey.

### Outcomes

We present analyses of three secondary outcomes nested within our larger survey study of partner engagement in CRC surveillance: 1) Discordance in communication about cancer (cancer communication) within dyads; 2) Discordance in experience of FCR within dyads; and 3) Survivors’ HrQoL.

### Measures

Measure development was based on a conceptual framework of couples dealing with cancer developed by Northouse et al. [21] and was informed by research by our team and others on the role of family and friends in decision making [22, 25]. We used standard techniques to assess content validity, including expert reviews and cognitive pretesting and pilot testing of measures in selected populations.

#### Communication

**Fig. 1:**
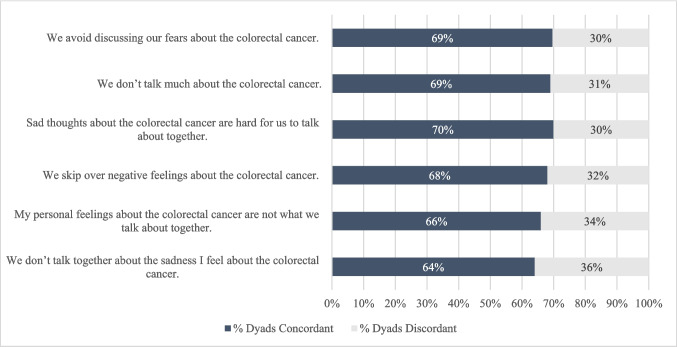
Communication items that evoked discordant responses in ≥ 30% of dyads

To measure cancer communication, we used the Lewis Mutuality and Interpersonal Sensitivity Scale (MIS), a 23- item self-reported questionnaire that was initially developed in the context of breast cancer patients and their family members, but has since been used to assess how couples communicate about cancer more broadly (baseline Cronbach alpha = 0.90 for patients, 0.91 for partners) [26, 27]. The MIS measures two domains relevant to dyadic communication: 1) Mutuality, defined as open communication within dyads; and 2) Interpersonal sensitivity, defined as the degree to which each member of the dyad elicits, attends to, and is aware of the other’s feelings and thoughts about the cancer. See Fig. 1 for representative items from the MIS.

#### Fear of cancer recurrence

**Fig. 2:**
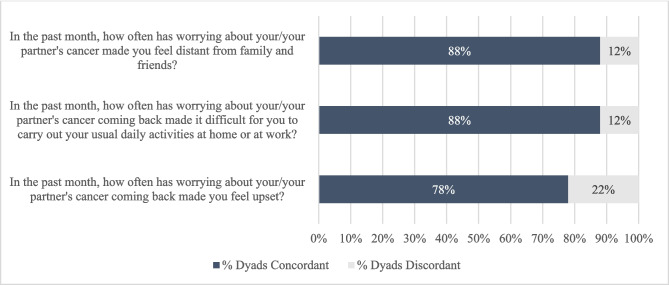
Fear of Recurrence Item-level Discordance

To measure their own experiences of FCR, we asked survivors and partners three questions, adapted from our prior work assessing FCR among more than 500 racially and ethnically diverse dyads of breast cancer survivors and their partners [28], about how often worry about recurrence has a negative psychosocial impact in their own lives (5-point Likert response scale from “Almost never” to “Almost always” with higher scores indicating greater FCR). Partners and survivors were asked the same questions, with each being asked about their own fear about the survivor’s cancer coming back. See Fig. 2 for representative items assessing FCR.

#### Health-related quality of life

We measured HrQoL using the PROMIS-29 + 2 Profile, version 2.1, a scale that is commonly used and has been extensively validated [29, 30]. Higher scores indicate better quality of life.

### Covariates 

Survivors and partners reported their age, sex, race and ethnicity, educational attainment (high school or less, some college, college graduate), and comorbid health conditions (0, ≥ 1). Because of observed co-linearity between partner and survivor sociodemographic factors, education was the only partner-reported covariate included in these analyses and was not highly co-linear. Survivors also reported annual household income (< $20,000, $20,000-$39,999, $40,000-$89,999, ≥ $90,000), and cancer treatment, including receipt of chemotherapy (yes/no) and radiation therapy (yes/no), as done in our prior work [31].

### Missing data 

In both survivor and partner surveys there were few missing values (< 3%) for all variables except income, for which 13% of survivors did not respond or reported they did not know. Multiple imputation techniques were used to account for the missing annual household income data [32, 33]. Five separate datasets containing plausible values for missing income were created using regression models with the SAS minianalyze procedure. Regression models were run separately on each dataset, and the results were combined using Rubin’s method [34].

### Statistical analysis

First, we examined discordance between survivor and partner responses to individual survey items pertaining to cancer communication and experiences of FCR. All items had a 5-point Likert response which we coded from 1–5. We created a discordance score, ranging from 0 to 4, of the absolute value of the difference between survivor and partner response for each item. A discordance score of “0” corresponds to complete agreement and a score of “4” corresponds to complete disagreement or discordance; i.e. if both survivor and partner answered “always true” to a statement, the discordance score is 0, while if survivor answered “always” and partner answered “never” (or vice versa), the discordance score is 4. Responses were then categorized as concordant (discordance score of 0 or 1), or discordant (discordance score ≥ 2). Then, for each survey item, we calculated the proportion of dyads with discordant responses.

Next, we examined discordance between survivor and partner for their composite responses to the cancer communication and FCR scales. We calculated the numeric average of responses to the individual items in each scale (i.e. cancer communication and FCR) and created a composite cancer communication response score and a composite FCR response score for each survivor and partner. We then calculated the absolute difference between survivor and partner composite response scores to create a continuous discordance score. For ease of interpretation, these discordance scores were standardized to have a standard deviation of 1 and were used in all subsequent bivariate analyses and multivariable logistic regressions. A higher discordance score indicates a greater degree of discordance. Additional details regarding this process are available in Appendix Table 1.

Then, we examined bivariate associations between the communication and FCR discordance scores and selected demographic and clinical covariates using ANOVA tests. We also investigated bivariate associations between survivors’ HRQoL and discordance in cancer communication and FCR using linear regressions. Finally, we estimated a generalized linear regression model to assess associations between survivors’ HRQoL and discordance in cancer communication and FCR, while adjusting for covariates.

## Results

### Survivor and partner characteristics 

Surveys were completed by 501 (51%) of 986 eligible survivors. Among those, 428 (85%) reported having a partner. Three hundred eleven partners returned surveys (73% partner response rate). Four partner surveys were returned without a corresponding completed survivor survey; therefore, paired surveys from 307 survivor-partner dyads were included in these analyses. Survivor response rates were significantly lower for non-White survivors, male survivors, and survivors identified through the Georgia Cancer Registry (all *p* < 0.05). Compared to survivors with a partner, unpartnered survivors were more likely to be female or Black (all *p* < 0.05).

Characteristics of survey respondents have been reported previously [19] and are shown in Table 1. Briefly, among survivors the mean age was 62.7 (standard deviation 11.3) years; 64% were male; 85% were white; 43% were college graduates; 38% reported annual household income ≥ $90,000; 93% reported receipt of chemotherapy; and 62% were 3–4 years out from diagnosis. Among partners, the mean age was 63.7 (standard deviation 11.1) years; 63% were female; 86% were white; and 37% were college graduates. 2% of dyads were in same-sex relationships. 86 dyads were recruited from University of Michigan, 4 from Billings Clinic, and 217 from the Georgia Cancer Registry.

**Table 1:** Characteristics of survivor (*n* = 307) and partner (*n* = 307) respondents

Characteristic	Survivors, *n* (%)	Partners, *n* (%)
Age (years) Mean (standard deviation) < 50 51–64 ≥65 Missing	62.7 (11.3) 36 (12%) 128 (42%) 142 (46%) 1 (0%)	63.7 (11.1) 48 (16%) 118 (40%) 130 (44%) 11 (4%)
Sex Male Female Missing	195 (64%) 111 (36%) 1 (0%)	113 (37%) 189 (63%) 5 (1%)
Race White Black Other	262 (85%) 23 (8%) 22 (8%)	263 (86%) 20 (7%) 24 (8%)
Educational attainment High school or less Some college College graduate Missing	70 (23%) 104 (34%) 133 (43%) 0 (0%)	82 (27%) 108 (35%) 114 (37%) 3 (1%)
Annual household income < $20,000 $20,000-$39,999 $40,000–89,999 ≥$90,000 Missing	21 (7%) 34 (11%) 95 (31%) 116 (38%) 41 (13%)	NA NA NA NA NA
Years since survivor diagnosis 1–2 3–4 ≥5 Missing	78 (26%) 182 (62%) 36 (12%) 11 (4%)	NA NA NA NA
Years partnered Mean (standard deviation)	33.9 (14.5)	NA
Receipt of Chemotherapy Yes No Missing	285 (93%) 16 (5%) 6 (2%)	NA NA NA
Receipt of Radiation Yes No Missing	102 (33%) 198 (65%) 6 (2%)	NA NA NA

### Cancer communication

The MIS yielded a high internal consistency reliability coefficient (Cronbach alpha = 0.935). The average proportion of discordant dyads for each of the 23 individual cancer communication items was 24% (range 9%−36%, standard deviation 8%). The communication items with the highest proportion of concordant responses were, “We keep communication open between us about the colorectal cancer,” (91% of dyads agreed or strongly agreed), “We understand how each of us is feeling about the colorectal cancer,” (88% agreed or strongly agreed), and “We try to support each other’s feelings about the colorectal cancer,” (88% agreed or strongly agreed). Six statements evoked discordant responses in at least 30% of dyads. These statements all focused on negative feelings, sadness, avoidance, or fear with respect to communication about the patient’s cancer (Fig. 1). 29% of dyads were discordant in their composite responses to the cancer communication scale. Discordance was seen in both directions—survivor reported better communication than partner in 16% of dyads, and partner reported better communication than survivor in 13% of dyads. Bivariate analyses are shown in appendix Table 2. After adjustment (Table 2), greater discordance in cancer communication was significantly associated with female sex of the survivor (*p* = 0.012) and partners having some college or college graduate level education (*p* = 0.030).

**Table 2:** Multivariable analyses of discordance in communication and fear of cancer recurrence

Characteristic	Discordance in communication		Discordance in fear of cancer recurrence	
	Mean discordance score (standard error)*	***p***	Mean discordance score (standard error)*	***p***
Age (per every 10 year decade)	0.07 (0.56)	0.366	−0.22 (0.08)	0.006
Sex Male Female	REF 0.33 (0.13)	0.012	REF 0.07 (0.13)	0.599
Race White Black Other	REF −0.11 (0.25) −0.07 (0.25)	0.887	REF 0.35 (0.25) −0.26 (0.26)	0.195
Survivor educational attainment High school or less Some college College graduate	REF 0.13 (0.17) −0.21 (0.18)	0.068	REF −0.28 (0.18) −0.16 (0.18)	0.303
Partner educational attainment High school or less Some college College graduate	REF 0.32 (0.17) 0.48 (0.18)	0.030	REF 0.17 (0.17) 0.17 (0.18)	0.587
Annual household income < $20,000 $20,000-$39,999 $40,000-$89,999 ≥$90,000	REF −0.04 (0.27) −0.32 (0.25) −0.20 (0.26)	0.368	REF −0.09 (0.27) −0.12 (0.25) −0.13 (0.26)	0.968
Years since survivor diagnosis 1–2 3–4 ≥5	REF −0.07 (0.14) 0.12 (0.22)	0.649	REF −0.02 (0.14) −0.14 (0.22)	0.805
Years partnered (per every 10 years)	−0.07 (0.06)	0.209	0.04 (0.06)	0.494
Receipt of chemotherapy No Yes	REF 0.26 (0.30)	0.387	REF −0.39 (0.31)	0.209
Receipt of radiation No Yes	REF −0.09 (0.14)	0.497	REF 0.26 (0.13)	0.049

*Coefficient from linear regression model. Outcome is standardized to have standard deviation = 1.0, and result can be interpreted as difference measured in standard deviations

### Fear of cancer recurrence

The FCR scale yielded a high internal consistency reliability coefficient (Cronbach alpha = 0.845). The average proportion of discordant dyads for each of the individual FCR items was 15% (range 12%−22%, standard deviation 4.4%; Fig. 2). The FCR item with the highest proportion of discordant responses (22% discordant responses) was, “In the past month, how often has worrying about your/your partner’s cancer coming back made you feel upset?” 26% of dyads were discordant in their composite responses to the FCR scale. Discordance was seen in both directions—survivor reported greater FCR than partner in 11% of dyads, and partner reported greater FCR than survivor in 15% of dyads. Bivariate analyses are shown in appendix Table 5. After adjustment (Table 2), greater discordance in FCR was significantly associated with younger survivor age (*p* = 0.006) and receipt of radiation therapy (*p* = 0.049).

### Health related quality of life

The PROMIS scale yielded a high internal consistency reliability coefficient (Cronbach alpha = 0.964). Bivariate analyses are shown in appendix Table 6. After adjustment (Table 3), worse survivor HRQoL was significantly associated with discordance in FCR (*p* < 0.001) and receipt of radiation therapy (*p* < 0.001). In a separate model evaluating the direction of discordance using a three-level discordance variable (survivor higher than partner vs. survivor and partner equal vs. partner higher than survivor), decreased survivor HRQoL was significantly associated with discordance in FCR in both directions, with a greater decrease in survivor HRQoL if the survivor had higher FCR than the partner (adjusted mean HRQoL 0.39) than if the partner had higher FCR than the survivor (adjusted mean HRQoL 0.88). There was no significant association between survivor HRQoL and discordance in communication (*p* = 0.640).

**Table 3:** Multivariable analyses of survivor Health-Related Quality of Life

Characteristic	Mean HRQoL (standard error)*	*P*
Discordance in communication	−0.03 (0.07)	0.640
Discordance in fear of recurrence	−0.32 (0.06)	< 0.001
Age (per every 10 years)	−0.01 (0.08)	0.918
Sex Male Female	REF −0.05 (0.13)	0.687
Race White Black Other	REF −0.10 (0.25) −0.24 (0.25)	0.599
Survivor Educational attainment High school or less Some college College graduate	REF −0.01 (0.18) 0.13 (0.18)	0.608
Partner Educational attainment High school or less Some college College graduate	REF −0.07 (0.17) −0.11 (0.18)	0.833
Annual household income < $20,000 $20,000-$39,999 $40,000–89,999 ≥$90,000	REF 0.33 (0.27) 0.29 (0.25) 0.52 (0.26)	0.196
Years since survivor diagnosis 1–2 3–4 ≥5	REF 0.13 (0.14) −0.06 (0.22)	0.485
Years partnered (per every 10 years)	−0.02 (0.05)	0.621
Receipt of chemotherapy No Yes	REF 0.05 (0.31)	0.860
Receipt of radiation No Yes	REF −0.48 (0.13)	< 0.001

*Coefficient from linear regression model. Outcome is standardized to have standard deviation = 1.0, and result can be interpreted as difference measured in standard deviations

## Discussion

In this cross-sectional dyadic survey of CRC survivors and their partners, we found that while most dyads were concordant in their responses to items assessing communication about the cancer and experiences of FCR, a substantial proportion of dyads were discordant in important components of communication and in FCR. Worse HRQoL among survivors was associated with greater discordance in FCR, but not with discordance in cancer communication. This investigation into the alignment of communication and shared fears between CRC survivors and their partners offers valuable insights that contribute to and expand upon the existing literature on the important and often under-recognized role of dyadic relationships in cancer survivorship and survivors’ HRQoL. Our finding that discordance in negative feelings about the cancer and fear may be more strongly associated with survivors’ HRQoL than discordance in communication more broadly is an important and novel contribution to the literature with implications for clinical care and future interventions.

Within the existing literature, open or concordant communication has been identified as a cornerstone for optimal relationship functioning among patients with cancer and their partners [13, 14]. Concordant communication allows each member of a dyad to understand the other’s worries and fears, check in regarding adverse effects of the cancer or its treatment, and coordinate coping mechanisms to better respond as a dyad to stressors as they arise. While most dyads in our study endorsed concordant responses to communication items that probed fundamental practices such as open communication and supporting each other’s feelings, there were numerous communication items that evoked discordant responses in a substantial proportion of dyads. Interestingly, the 6 communication items that evoked the greatest proportion of discordant responses were all negative statements, centered on themes of sadness, avoidance, and fear related to the cancer. While such breakdowns in communication could potentially contribute to distress they could also be an example of protective buffering, where one member of the dyad avoids discussing negative feelings to shield the other from added distress [14, 35].

Prior research has linked open or concordant communication with improved dyadic coping and resilience [36, 37]. Therefore, our finding that survivors’ HRQoL was not associated with discordance in cancer communication was unexpected. One possible explanation is that it is not the quality or concordance of communication, but the nature and content of what is being communicated, that might have more impact on quality of life. For example, our finding that worse HRQoL among survivors was associated with greater discordance in FCR is well-aligned with the existing literature. FCR has been reported to be common among CRC survivors [5], persist years into survivorship, and negatively impact survivors’ quality of life [6, 7, 38]. While FCR has been described among CRC survivors of all ages, several studies suggest it may be a particular concern among younger survivors [6, 39, 40]; we also found that greater discordance in FCR was associated with younger survivor age (co-linear with partner age). FCR affects partners as well—previous studies of cancer survivors and their families found that partners and family caregivers were just as likely, or more likely, than survivors to report fear of the cancer recurring [9, 11]. Notably, we found that while decreased survivors’ HRQoL was associated with discordance in FCR in both directions (e.g., survivor had higher FCR than partner or partner had higher FCR than survivor), the decrease in HRQoL was greater when the survivor had higher FCR than the partner. This finding suggests that both individual-level interventions focused on the survivor, as well as dyadic interventions that include the partner, may be needed to address FCR and improve HRQoL.

The CRC survivorship period presents unique opportunities to identify and intervene upon issues related to dyadic communication and FCR. Because CRC survivors undergo a five-year regimen of surveillance care to detect cancer recurrence following completion of curative-intent therapy, they maintain frequent contact with the health care system. Undergoing clinical exams, blood tests and imaging studies as frequently as every three to six months may increase negative feelings about the cancer and FCR among CRC survivors—in a 2015 study of CRC survivors, medical examinations and appointments with doctors were among the most frequently reported triggers of FCR [7]. However, these frequent contacts with the clinical care team also provide ideal opportunities to ask survivors about concerns related to dyadic communication about the cancer and FCR. As our findings highlight, clinicians should recognize that even survivor-partner dyads that appear to communicate openly may require additional probing and support to address issues related to negative feelings and fear; referrals to social work and psychology may be appropriate and helpful for many dyads. Although it may seem that addressing the needs of partners is outside the locus of control of the oncologist, recognizing the importance of dyadic communication and relationship functioning to patients’ HRQoL is a central tenet of patient- and family-centered cancer care [41, 42].

Over the past two decades there have been numerous studies of dyadic communication interventions for couples dealing with a cancer diagnosis. These interventions typically focus on the period of time shortly after diagnosis or during active cancer treatment, with some focusing on patients living with metastatic cancer. They have been developed in multiple formats—In-person, telephone, and web-based—and have been shown to improve outcomes across multiple domains, including physical health, psychological health, quality of life, and dyadic relationship [43, 44]. In addition, there exist a number of web-based interventions for cancer survivors and their caregivers. These interventions tend to focus more on individual-level coping (e.g., symptom management, self-care strategies, psychotherapy etc. tailored to survivors and caregivers individually) and are less likely to include training in dyadic communication and coping or assess dyadic outcomes [45]. Therefore, there is a need for the development of targeted interventions to help survivors and their partners communicate about survivorship concerns, including FCR. A number of therapeutic interventions for FCR have been tested across cancer types, and those that incorporate acceptance and commitment therapy, cognitive behavioral therapy, and mindfulness-based therapeutic interventions have shown long-term benefits [46]. Future studies are needed to assess the impact of interventions that provide these therapeutic approaches to each individual in a dyad together with dyadic communication training.

There were several limitations to our study that warrant mention. Although we enrolled a random sample of male and female survivors from a large academic cancer center, a rural community oncology practice, and the population-based Georgia Cancer Registry (which includes the entire state of Georgia), the cohort lacked racial and ethnic diversity as well as dyads outside of heteronormative relationships. We were not able to distinguish between dyads who were married vs. partnered and cohabitating, and we did not collect data about whether dyads had children. We do not know whether partners had cancer themselves, which is a potential unmeasured confounder. As with all survey studies, nonresponse bias was possible. When compared with survivors whose partners responded to the survey, survivors whose partners did not respond were more likely to be female and Black. Our results may underestimate dyadic discordance because dyads who responded to our survey may have been more likely to communicate openly about the cancer compared to those who did not respond. While we did not use an externally validated measure of FCR, we used a scale used in our prior work to assess FCR among more than 500 racially and ethnically diverse dyads of breast cancer survivors and their partners [28] and the scale yielded a high internal consistency reliability coefficient in our current study. We also note that our study was cross-sectional. In the future, longitudinal studies that use externally validated measures of FCR such as the Cancer Worry Scale [47] or the Fear of Cancer Recurrence Inventory [48] may help elucidate changes in dyadic communication and FCR that occur throughout the survivorship period.

## Conclusion

Our study underscores the multifaceted nature of dyadic experiences throughout CRC survivorship. While open communication remains pivotal, our findings suggest that even dyads that appear to communicate openly may be discordant in their negative feelings and fear about the cancer, and that discordance in shared fears and concerns may influence HRQoL more directly. Addressing these shared concerns, especially FCR, can pave the way for patient-centered survivorship care, enhancing both the quality of life of survivors and the strength of their partnerships.

## Data Availability

Data are available upon reasonable request to the authors.

## Appendix

**Table 4:** Item-level Discordance Scores

	**Survivor Response**				
**Partner Response**	Almost never	Rarely	Sometimes	Often	Almost always
Almost never	Discordance score 0	Discordance score 1	Discordance score 2	Discordance score 3	Discordance score 4
Rarely	Discordance score 1	Discordance score 0	Discordance score 1	Discordance score 2	Discordance score 3
Sometimes	Discordance score 2	Discordance score 1	Discordance score 0	Discordance score 1	Discordance score 2
Often	Discordance score 3	Discordance score 2	Discordance score 1	Discordance score 0	Discordance score 1
Almost always	Discordance score 4	Discordance score 3	Discordance score 2	Discordance score 1	Discordance score 0

For both communication about cancer and FCR, discordance scores were calculated for each individual item in the measure by comparing the survey response selected by the survivor to the survey response selected by the partner. A discordance score ranging from 0 (complete concordance) to 4 (complete discordance) was assigned as per the table. Item level responses with a discordance score of 0 or 1 were categorized as concordant. Item level responses with a discordance score ≥ 2 were categorized as discordant.

We then created composite discordance scores for use in the bivariate and multivariable analyses. We first calculated the numeric average of each survivor’s and partner’s responses to the individual items in the communication about cancer and the FCR scales. Each individual item had a 5-point Likert response and the responses were assigned a numeric value from 1–5—almost never to almost always—on the Likert scale. We then created a composite Communication about cancer response score and a composite FCR response score for each individual survivor and partner. For example, the FCR measure consists of 3 items each with a 5-point Likert response. If the respondent selects “almost never” (1 point) for all 3 items, the composite FCR response score is the numeric average of those responses or (1 + 1 + 1)/3 = 1. Next, among paired survivor-partner dyads we calculated the absolute difference between survivor and partner composite response scores and used those to create a continuous discordance score for Communication about cancer and a continuous discordance score for FCR. For example, if a survivor’s composite FCR response score was 5 and the partner’s composite FCR response score was 1, the composite FCR discordance score would be 4. We standardized these discordance scores to have a standard deviation of 1 for ease of interpretation. We used the standardized discordance scores in all subsequent bivariate and multivariable logistic regressions. A higher discordance score indicates a greater degree of discordance.

**Table 5:** Bivariate analyses of discordance in communication and fear of recurrence

Characteristic	Discordance in Communication		Discordance in fear of recurrence	
	**Mean discordance score (standard error)**	***p***	**Mean discordance score (standard error)**	***p***
Survivor Age (years) ≤ 50 51–64 ≥65	12.20 (1.99) 12.52 (0.92) 12.39 (0.94)	0.99	1.06 (0.16) 0.65 (0.06) 0.56 (0.05)	< 0.01
Survivor Sex Male Female	11.02 (0.76) 14.88 (1.04)	< 0.01	0.65 (0.05) 0.68 (0.06)	0.71
Survivor Race White Black Other	12.44 (0.65) 12.11 (3.14) 12.26 (2.50)	0.99	0.64 (0.04) 0.89 (0.22) 0.59 (0.10)	0.26
Survivor Educational attainment High school or less Some college College graduate	10.66 (1.07) 14.77 (1.29) 11.47 (0.83)	0.02	0.67 (0.09) 0.64 (0.07) 0.66 (0.05)	0.96
Partner Educational attainment High school or less Some college College graduate	10.41 (1.16) 12.91 (1.07) 13.44 (1.01)	0.14	0.59 (0.06) 0.68 (0.07) 0.66 (0.06)	0.65
Annual household income < $20,000 $20,000-$39,999 $40,000-$89,999 ≥$90,000	13.05 (1.89) 14.06 (2.40) 12.54 (1.06) 13.17 (1.03)	0.92	0.79 (0.13) 0.63 (0.13) 0.66 (0.07) 0.70 (0.06)	0.82
Years since survivor diagnosis 1–2 3–4 ≥5	13.26 (1.38) 11.61 (0.71) 14.34 (2.12)	0.27	0.70 (0.08) 0.65 (0.05) 0.59 (0.10)	0.71

**Table 6:** Bivariate analyses of Survivor Health-Related Quality of Life

Characteristic	Mean HRQoL (standard error)	*P*
Discordance in Communication	−0.04 (0.06)	0.551
Discordance in Fear of Recurrence	−0.29 (0.06)	< 0.01
Survivor Age (years) ≤ 50 51–64 ≥65	−0.01 (0.16) 0.02 (0.10) 0 (0.08)	0.98
Survivor Sex Male Female	0.03 (0.07) −0.02 (0.09)	0.69
Survivor Race White Black Other	0.06 (0.06) −0.13 (0.24) −0.52 (0.22)	0.04
Survivor Educational attainment High school or less Some college College graduate	−0.06 (0.13) −0.12 (0.10) 0.14 (0.09)	0.12
Partner Educational attainment High school or less Some college College graduate	−0.04 (0.12) 0.01 (0.09) 0.07 (0.10)	0.77
Annual household income < $20,000 $20,000-$39,999 $40,000–89,999 ≥$90,000	−0.49 (0.21) −0.19 (0.15) −0.03 (0.10) 0.20 (0.10)	0.01
Years since survivor diagnosis 1–2 –4 ≥5	−0.06 (0.11) 0.04 (0.08) −0.02 (0.19)	0.76
